# Human* Papilloma Virus* Infection in Patients with Male Accessory Gland Infection: Usefulness of the Ultrasound Evaluation

**DOI:** 10.1155/2016/9174609

**Published:** 2016-05-03

**Authors:** Rosita A. Condorelli, Enzo Vicari, Laura M. Mongioi, Giorgio I. Russo, Giuseppe Morgia, Sandro La Vignera, Aldo E. Calogero

**Affiliations:** ^1^Department of Clinical and Experimental Medicine, University of Catania, 95123 Catania, Italy; ^2^Department of Urology, University of Catania, 95123 Catania, Italy

## Abstract

This study evaluated the ultrasound (US) features of 20 patients with MAGI and concomitant* papilloma virus* (HPV) infection compared to 20 patients with microbial (presence of* Chlamydia trachomatis* alone) MAGI and 20 patients with amicrobial (inflammatory) MAGI. Patients with HPV infection showed significantly (*p* < 0.05) higher total prostate, seminal vesicles, and epididymal US signs (18.0 ± 2.0) compared to the other 2 groups (12.0 ± 4.0 versus 10.0 ± 3.0, resp.). In addition, patients with MAGI and HPV had a higher prevalence of complicated forms of MAGI [prostatovesiculitis (PV) and prostate-vesiculo-epididymitis (PVE)] and a higher frequency of the fibrosclerotic variant compared to the other groups (70.0 ± 10.0% versus 48.0 ± 5.0% versus 15.0 ± 10.0%). Moreover, HPV infected patients had a higher number of US criteria suggestive for MAGI in the periurethral region of the prostate compared to the other groups. In particular, the patients showed a higher ratio between periurethral and lobar US criteria distribution (5.0 versus 0.5). Finally, the seminal fluid concentration of CD45_pos_ leukocytes (2.0 ± 0.2 versus 1.3 ± 0.3 versus 1.0 ± 0.3 mil/mL) was significantly higher and sperm progressive motility was significantly lower in these patients compared to other groups.

## 1. Introduction

Male accessory gland inflammation/infection (MAGI) represents an important cause of male infertility. The first definition was suggested in 1980 [1]. Subsequently, in 1993, the World Health Organization proposed the first diagnostic algorithm [2]. Currently the European Association of Urology guidelines confirmed this condition among the potential causes of male infertility [3].

For several years, we have investigated different aspects of this condition, in particular, the problem of the epidemiological conflicting data [4–6], the different pathophysiological mechanisms associated with the alterations of the main sperm parameters, the symptomatic characterization of the patients, the different laboratory tests to improve the diagnosis of this condition, and the differences between microbial and inflammatory forms. From the biofunctional point of view we have previously seen that this pathological condition is able to alter some important unconventional parameters, such as fragmentation of sperm DNA, mitochondrial membrane potential, quality of chromatin compaction, and the degree of sperm apoptosis [7]. Recently, we reported a high frequency of human* papilloma virus*- (HPV-) DNA in the semen of these patients [8].

Another aspect that we extensively explored is the sex accessory gland US characterization in patients with MAGI. In particular, we have shown that US evaluation is of fundamental relevance to understand the anatomical site of inflammation [prostatitis (P), prostatovesiculitis (PV), and prostate-vesiculo-epididymitis (PVE)], the side of involvement (unilateral versus bilateral forms), the predictive criteria of clinical response to pharmacological treatment (lower in PV and PVE compared to P), and the different prognostic value of some US categories (fibrosclerotic and hypertrophic-congestive forms), with the possibility for the clinical andrologist to improve the diagnostic workup, monitoring and evaluating the effectiveness of the specific treatment [8–14]. The diagnostic specificity of US scans in patients with MAGI was recently confirmed by other studies, which evaluated the relationship between US criteria and some laboratory abnormalities, particularly the seminal concentrations of interleukin 8, considered one of the main markers of chronic inflammation [15]. Even the symptomatology of these patients has been shown to relate to their US appearance [16].

This study was undertaken to compare prostate, seminal vesicles, and epididymal US characteristics in patients with inflammatory, bacterial (*Chlamydia trachomatis*), and viral (HPV) related MAGI. To accomplish this, the following parameters were taken into consideration: total number of US abnormalities, frequency of uncomplicated (P) versus complicated forms (PV and PVE) of MAGI, unilateral versus bilateral forms, and fibrosclerotic versus hypertrophic-congestive forms. Another end-point of this study was to evaluate the possible differences on the seminal concentration of peroxidase-positive and CD45_pos_ leukocytes among these three groups.

## 2. Patients and Methods

### 2.1. Patient Selection

We evaluated 60 consecutive patients with MAGI according to the diagnostic criteria suggested by the WHO [2] as follows.

*Clinical and Ultrasound Criteria Used for the Diagnosis of Male Accessory Gland Infection/Inflammation*

 Clinical criteria are the following:
   
*Criterion A*: history of urogenital infection and/or abnormal rectal palpation.
   
*Criterion B*: significant alterations in the expressed prostatic fluid and/or urinary sediment after prostatic massage.
   
*Criterion C*:
    (C1) uniform growth of more than 10^3^ pathogenic bacteria or more than 10^4^ nonpathogenic bacteria per mL, in culture of diluted seminal plasma,
    (C2) presence of more than 10^6^ peroxidase-positive leucocytes per mL of ejaculate,
    (C3) signs of disturbed secretory function of the prostate or seminal vesicles.
   The diagnosis was made when at least one of the following criteria combinations was found:
    (i) A + B,
    (ii) A + C (1, 2, or 3),
    (iii) B + C (1, 2, or 3),
    (iv) C1 + C2,
    (v) C1 + C3,
    (vi) C2 + C3.
 Prostatitis is suspected in the presence of >2 of the following ultrasound signs:
  1. Asymmetry of the gland volume.
  2. Areas of low echogenicity.
  3. Areas of high echogenicity.
  4. Dilatation of periprostatic venous plexus.
  5. Single or multiple internal cystic-like areas.
  6. Area/s of moderate increased vascularity (focal or multiple).
 Vesiculitis is suspected in the presence of >2 of the following ultrasound signs:
  1. Increased (>14 mm) mono- or bilateral anteroposterior diameter.
  2. Asymmetry >2.5 mm (normal 7–14 mm) compared to the contralateral vesicle.
  3. Reduced (<7 mm) mono- or bilateral anteroposterior diameter.
  4. Thickened and/or calcified glandular epithelium.
  5. Polycyclic areas separated by hyperechoic septa in one or both vesicles.
  6. Fundus/body ratio >2.5.
  7. Fundus/body ratio <1.
  8. Anteroposterior diameter unchanged after ejaculation.
 Epididymitis is suspected in the presence of >2 of the following ultrasound signs:
  1. Mono- or bilateral increased size of the head (craniocaudal diameter >12 mm) and/or of the tail (craniocaudal diameter >6 mm).
  2. Mono- or bilateral presence of multiple microcystis in the head and/or tail.
  3. Mono- or bilateral low echogenicity or high echogenicity.
  4. Mono- or bilateral large hydrocele.
  5. Enlargement of the superior part of the cephalic tract and superior/inferior portion ratio >1.
  6. Unchanged anteroposterior diameter of the tail after ejaculation.

The patients were further assessed for the presence of microbes or HPV in their seminal fluid. On the basis of these results, we selected 20 consecutive patients with MAGI and HPV, 20 patients with MAGI and* Chlamydia trachomatis*, and 20 patients with amicrobial MAGI (classified as inflammatory MAGI) [8, 14].

During the first consultation, all patients underwent a specific questionnaire to evaluate their symptoms [17]. The structured interview about MAGI (SI-MAGI) is structured into four domains (urinary tract symptoms, ejaculatory pain or discomfort, sexual dysfunction, and quality-of-life impact) for a total of 30 questions with four possible answers.

Then, all underwent didymo-epididymal and prostate-vesicular US scans, according to the US criteria previously published [11] (see the diagnostic criteria shown above). US evaluation was carried out twice for each patient, by two operators who had received a similar training on the US criteria for MAGI diagnosis. The evaluation of the epididymal region was performed along various longitudinal, transverse, and oblique scans with the patient lying in a supine position using a 7.5 MHz high-frequency linear probe (6–13 MHz). The prostate-vesicular region was evaluated by transrectal ultrasonography through transverse, longitudinal, and oblique scans with patients placed in the left lateral decubitus. A transrectal biplanar probe (i.e., linear transducer 7.5 MHz; convex transducer 6.5 MHz) with an “end fire” transducer was used.

*Chlamydia trachomatis* and HPV infection were evaluated as previously described [8, 14].

### 2.2. Ultrasound End-Points Evaluated

The following US parameters were evaluated in the 3 groups of patients with MAGI: (1) total number of US criteria suggestive for MAGI; (2) total number of US criteria in the prostate periurethral zone; (3) total number of US criteria in the prostate lobar zone; (4) US frequency of P; (5) US frequency of PV; (6) US frequency of PVE; (7) US frequency of monolateral P; (8) US frequency of monolateral PV; (9) US frequency of monolateral PVE; (10) US frequency of bilateral P; (11) US frequency of bilateral PV; (12) US frequency of bilateral PVE; (13) US frequency of fibrosclerotic form; and (14) US frequency of hypertrophic-congestive form.

**Figure 1:**
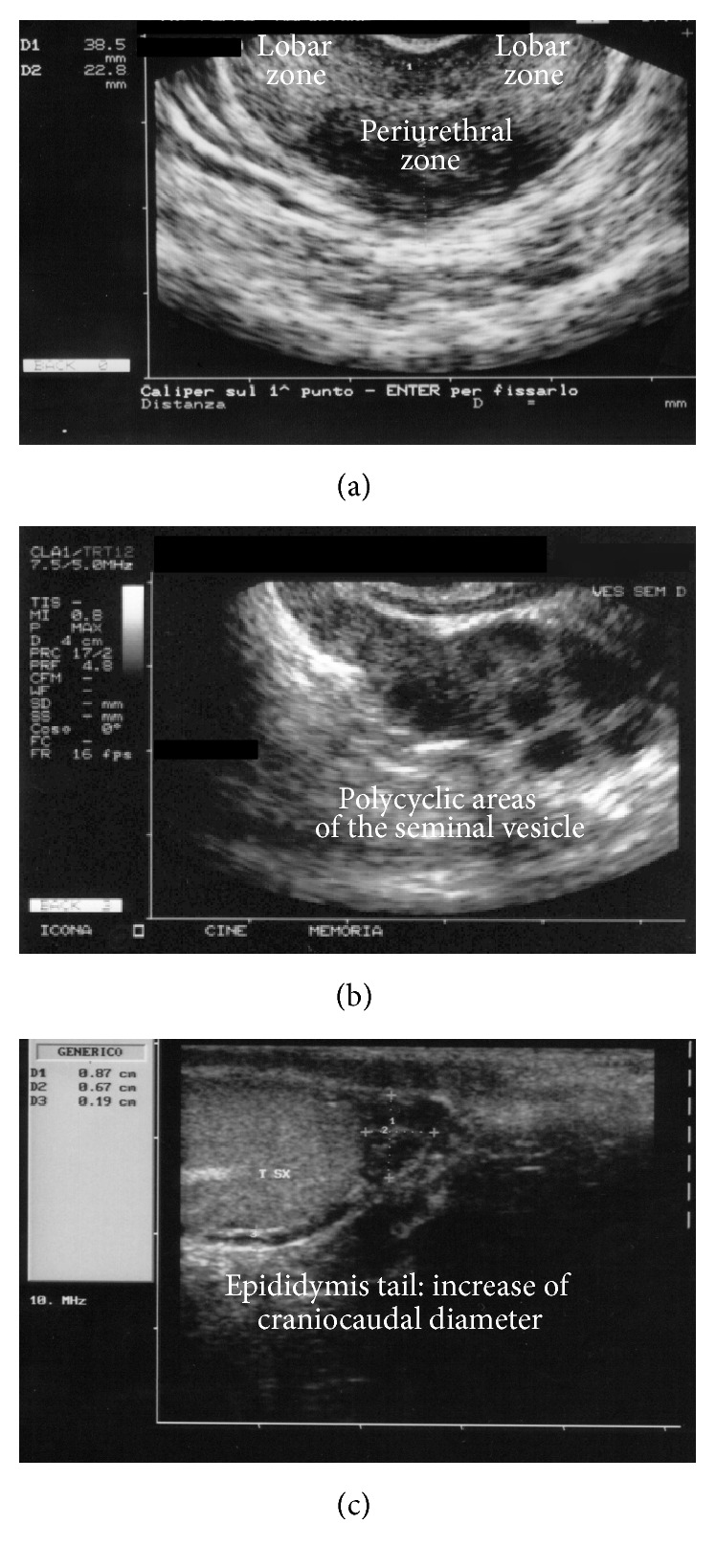
Examples of ultrasonographic suggestive images in patients with MAGI. (a) US subdivision of the prostate in periurethral and lobar regions. (b) Chronic vesiculitis. (c) Chronic epididymitis.

The periurethral region was considered as the intermediate portion of the transverse diameter of the prostate equally divided into three thirds.  Figure 1 illustrates some examples of ultrasonographic suggestive findings.

### 2.3. Sperm Analysis

Semen samples were collected by masturbation into a sterile container after 2–7 days of sexual abstinence and conveyed to the laboratory within 30 minutes after ejaculation. Each sample was evaluated for seminal volume, pH, viscosity, sperm count, progressive motility, morphology, and leukocytes concentration according to the WHO criteria [18]. Smears were fixed and stained with Papanicolaou stain.

### 2.4. Seminal Fluid Leukocyte Flow Cytometric Analysis

To carry out the absolute leukocyte count, 100 *μ*L of liquefied semen sample was incubated with a mixture containing Syto-16 green fluorescent nucleic acid stain to identify the spermatozoa and exclude debris (final concentration 200 nM, Molecular Probes, Eugene, Oregon, USA), 7-amino-actinomycin D (7-AAD Via-Probe, BD Pharmingen, San Diego, CA, USA) to assess viability, anti-CD45-APC (pan-leukocyte antigen) to recognize white blood cells, and anti-CD16-PE for PMN recognition. The addition of 100 *μ*L of Flow-Count*™* Fluorospheres (Beckmann-Coulter, Fullerton, CA, USA) at 1034 beads/mL allowed the determination of the absolute leukocyte count by flow cytometry. After incubation in the dark for 20 min at room temperature, 1 mL phosphate buffered saline was added, and the sample was analyzed by flow cytometry (EPICS XL Flow Cytometer, Coulter Electronics, IL, Italy). For each test, 100,000 events were acquired.

The study was approved by the Internal Institutional Board and all examined patients signed informed consent to the processing of personal data.

### 2.5. Statistical Analysis

Results are reported as mean ± SEM throughout the study. The data were analyzed by 1-way analysis of variance (ANOVA) followed by Tukey's test, as appropriate. The software SPSS 22.0 for Windows was used for statistical evaluation (SPSS Inc., Chicago, IL, USA). A statistically significant difference was accepted when the *p* value was lower than 0.05.

## 3. Results

Mean age and body mass index did not show significant differences among groups. Similarly, the scores of the questionnaire for the evaluation of the symptoms resulted in no statistical difference in the groups of patients studied (Table 1).

Seminal fluid volume, sperm concentration, and the percentage of spermatozoa with normal morphology did not vary significantly among the 3 groups of patients with MAGI. The percentage of spermatozoa with progressive motility was significantly lower in patients with MAGI HPV-positive compared to patients with microbial or amicrobial MAGI. Sperm progressive motility did not show any significant difference between these 2 latter groups. Similarly, the concentration of CD45_pos_ leukocytes in the seminal fluid was significantly higher in patients with HPV infection compared to the other groups, whereas peroxidase-positive leukocyte concentrations were similar in the 3 groups of patients. Finally the percentage of CD45_pos_-CD16_pos_ leukocytes was significantly lower in patients with HPV infection compared to patients with* Chlamydia* infection (Table 2).

At the US evaluation, patients with HPV infection showed a total number of US criteria suggestive for MAGI significantly higher compared to the other 2 groups. Moreover, these patients showed a higher frequency of bilateral PVE and fibrosclerotic variant compared to the other groups (Table 3). Finally patients with HPV infection had a higher number of US criteria suggestive for MAGI in the periurethral region of the prostate and a lower frequency of US criteria in the lobar region of the prostate compared to the other groups (Table 3). Consequently, patients with HPV infection showed a higher ratio between periurethral and lobar US criteria distribution (5.0 versus 0.5).

## 4. Discussion

The results of this study conducted on patients with MAGI, arbitrarily divided into three groups: viral (presence of HPV-DNA), microbial (presence of* Chlamydia trachomatis* alone), and amicrobial (inflammatory), suggest that, from the US point of view, patients with HPV infection have a significantly higher frequency of complicated forms of MAGI, represented by both PV and PVE. Moreover, these patients showed a higher prevalence of the fibrosclerotic variant compared to other groups. Finally, patients with MAGI and HPV showed a distribution of US feature suggestive for MAGI predominantly in the periurethral region of the prostate. From the clinical point of view, these results represent an important element of further evaluation relatively to the pathophysiological and prognostic significance of these three different aspects.

Although patients with MAGI are overall considered a high-risk category for male infertility, within this condition, bilateral PVE and the fibrosclerotic variant represent the categories of MAGI associated with lower sperm quality [9, 10]. Moreover, the complicated forms of MAGI (PV and PVE) represent the categories associated with lower clinical response (eradication) after pharmacological treatment [19]. Finally, the predominant prostate periurethral region localization of the US criteria suggestive for MAGI may represent a US sign that may be useful in the differential diagnosis with MAGI of other causes (microbial and amicrobial).

To our knowledge, no other clinical studies have evaluated the US features of patients with MAGI and HPV infection. In a previous study, we showed that the prevalence of HPV infection in patients with microbial MAGI was ≈30%, whereas the prevalence decreased to ≈20% among patients with inflammatory MAGI. Moreover, 40% of patients with MAGI and HPV infection had also* Chlamydia trachomatis* coinfection. In our experience, this represents the most common coinfection found in these patients [8]. The results of the present study confirm that patients with MAGI and HPV infection have a significant reduction in the percentage of spermatozoa with progressive motility as previously reported [20].

Although Krause and colleagues suggested a possible viral pathogenesis of the leukocytospermia [21], the evaluation of a possible viral infection in patients with increased seminal fluid leukocyte concentration is an uncommon practice. The results of this study suggest that the measurement of seminal fluid leukocytes using a peroxidation method has many limitations in the presence of viral infection, at least related to HPV. Indeed, the method suggested by the WHO manual [18] has the limitation to evaluate only the neutrophils, but not the lymphocytes eventually present in the semen. Hence, it is of no help for evaluating the effects of a chronic inflammatory response in patients who are often asymptomatic or paucisymptomatic and the use of the questionnaire is not useful to discriminate the etiological diagnosis. In contrast, the evaluation of seminal fluid leukocytes using an antibody against the pan-leukocyte CD45 antigen (and flow cytometry) has been shown capable of detecting the presence of leukocytospermia both in the present study and as previously reported [8]. Therefore, the optimal clinical management of patients with MAGI and HPV infection should include two additional aspects: an appropriate laboratory method to evaluate the inflammatory response of the semen with the evaluation of the CD45 leukocytes concentration and the US scan evaluation.

The predominat prostate periurethral distribution of the US criteria suggestive for MAGI in patients with HPV infection represents an unusual aspect that deserves further investigation. Urethra is the main structure where to search for HPV in men [22]. In addition, from the anatomical point of view, the preprostatic urethra is the most proximal portion of the posterior urethra which is about 1 cm long and extends from the internal urethral orifice at the entrance of the urethra into the prostate. The prostatic urethra is the portion of the urethra, 3-4 cm long, which runs within the prostate, close to the front face, and ends at the apex of the prostate. The most important feature of this stretch of the urethra is the urethral crest, a relief covered by mucous membrane that protrudes into the lumen starting from its rear wall, causing it to become arcuate in cross section. About halfway along the urethral crest there is a prominence called verumontanum or seminal colliculus which is perforated by an opening called prostatic utricle. The prostatic utricle is a small indentation located in the prostatic urethra, on the peak of the urethral crest, on the seminal colliculus (verumontanum), flanked by the openings of the ejaculatory ducts. Probably the canalicular backscatter of the infection justifies this distribution of US criteria predominantly in the periurethral region of the prostate.

The results of the present study represent a further confirmation for the evaluation of the possible presence of HPV infection and of the US scan usefulness in the patients with suspected MAGI. In particular, a possible viral etiology should be considered for all patients with some US scan peculiar findings: bilateral PVE, fibrosclerotic variant, and prevalent periurethral distribution of US signs in the prostatic region (Table 4). The symptoms, as shown in this study, are not able to discriminate between inflammatory and microbial forms. However, recent data of the literature showed an elevated frequency of HPV-DNA detection in cryopreserved semen samples that represent an important demonstration of the absence of an adequate clinical evaluation of patients, especially when they require gaining access to assisted reproductive techniques [23]. Moreover, the possible vaccination of these patients modifies the therapeutical approach, with the possibility of stimulating their immune response and to facilitate the clearance of the virus [24]. The persistent elevated seminal concentration of CD45-positive leukocytes may represent an important sign of active inflammatory response, also after adequate pharmacological treatment. This is a very important potential element for the possible consequences on sperm parameters. In fact, MAGI affects male fertility through four main mechanisms: anatomical obstruction of the seminal tract, altered secretory capacity of accessory glands, microbial direct effects, and finally the production of free oxygen radicals and/or cytokines [10].

In conclusion, the results of this study showed that patients with MAGI and HPV infection have a peculiar accessory sex gland US pattern consisting of a higher total number of features suggestive of MAGI particularly in the periurethral region of the prostate compared to patients with microbial (*Chlamydia trachomatis*) and amicrobial MAGI. Moreover, they have also a higher prevalence of bilateral PVE and of the fibrosclerotic variant which have a greater negative impact on sperm parameters. Accordingly, the presence of HPV-DNA is associated with a lower sperm progressive motility compared to other forms of MAGI. Finally, the HPV presence is associated with a leukocytospermia not detectable by the standard peroxidase method suggested by WHO [18]. Therefore, we suggest that didymo-epididymal and prostate-vesicular ultrasound scans and seminal fluid pan-leukocyte measurement should be used when a viral (at least in the case of HPV) etiology of MAGI is suspected.

## Figures and Tables

**Table 1:** Anthropometric measurements and clinical symptom scores in the three groups of patients with male accessory gland infection/inflammation (MAGI) enrolled in this study.

Parameters	MAGI HPV-positive	MAGI Chlamydia-positive	Inflammatory MAGI
Anthropometric parameters			
Age (years)	26.0 ± 4.0	26.0 ± 8.0	28.0 ± 6.0
BMI (kg/m^2^)	21.0 ± 4.0	20.0 ± 3.0	22.0 ± 4.0

Clinical symptoms/signs			
Urinary tract symptoms	8.0 ± 4.0	6.0 ± 3.0	6.0 ± 2.0
Spontaneous and/or ejaculatory pain or discomfort	14.0 ± 4.0	13.0 ± 4.0	11.0 ± 3.0
Sexual dysfunction	15.0 ± 4.0	14.0 ± 3.0	12.0 ± 6.0
Impact on the quality-of-life	12.0 ± 4.0	12.0 ± 2.0	10.0 ± 2.0

Urinary tract symptoms severity score: mild: 0–6; moderate: 7–12; severe: 13–18.

Spontaneous and/or ejaculatory pain or discomfort severity score: mild: 0–8; moderate: 9–16; severe: 17–24.

Sexual dysfunction severity score: mild: 0–11; moderate: 12–22; severe: 23–33.

Impact on the quality-of-life severity score: mild: 0–5; moderate: 6–10; severe: 11–15.

**Table 2:** Sperm parameters and seminal fluid leucocyte concentration measured by flow cytometry in the three groups of patients with male accessory gland infection/inflammation (MAGI) enrolled in this study.

Parameters	MAGI HPV-positive	MAGI Chlamydia-positive	Inflammatory MAGI
Seminal fluid volume (mL)	1.5 ± 0.5	1.8 ± 0.8	1.6 ± 0.8
Sperm concentration (million/mL)	11.0 ± 4.0	12.0 ± 4.0	14.0 ± 6.0
Progressive motility (%)	8.0 ± 3.0^*∗*†^	13.0 ± 5.0	15.0 ± 8.0
Normal forms (%)	4.0 ± 1.0	4.0 ± 2.0	5.0 ± 2.0
Peroxidase-positive leukocytes (million/mL)	2.4 ± 0.5	2.3 ± 0.8	2.0 ± 0.6
CD45_pos_ leukocytes (million/mL)	2.0 ± 0.2^*∗*†^	1.3 ± 0.3	1.0 ± 0.3
CD45+CD16+ (%)	23.0 ± 7.0^*∗*^	55.0 ± 12.0	50.0 ± 6.0

HPV: human *papilloma virus*. ^*∗*^ *p* < 0.05 versus patients with MAGI *Chlamydia trachomatis*-positive; ^†^ *p* < 0.05 versus patients with inflammatory MAGI.

**Table 3:** Ultrasound features in the three groups of patients with male accessory gland infection/inflammation (MAGI) enrolled in this study.

Parameters	MAGI HPV-positive	MAGI Chlamydia-positive	Inflammatory MAGI
Ultrasound (US) features			
Total number of US criteria suggestive for MAGI	18.0 ± 2.0^*∗*†^	12.0 ± 4.0	10.0 ± 3.0
Total number of US criteria in the prostate periurethral zone	5.0 ± 1.0^*∗*†^	2.0 ± 1.0	2.0 ± 1.0
Total number of US criteria in the prostate lobar zone	1.0 ± 1.0^*∗*†^	4.0 ± 1.0	4.0 ± 1.0

Prevalence of the various diagnostic categories of MAGI			
Prostatitis (%)	18.0 ± 6.0^†^	27.0 ± 5.0^†^	65.0 ± 10.0
Prostate-vesiculitis (%)	40.0 ± 10.0^†^	45.0 ± 5.0^†^	20.0 ± 5.0
Prostate-vesiculitis-epididymitis (%)	42.0 ± 4.0^*∗*†^	28.0 ± 5.0^†^	15.0 ± 5.0

Prevalence of monolateral versus bilateral forms of MAGI			
Monolateral prostatitis (%)	10.0 ± 3.0^*∗*†^	17.0 ± 5.0^†^	45.0 ± 5.0
Monolateral prostate-vesiculitis (%)	10.0 ± 5.0	15.0 ± 5.0	15.0 ± 5.0
Monolateral prostate-vesiculitis-epididymitis (%)	12.0 ± 5.0	10.0 ± 5.0	10.0 ± 5.0
Bilateral prostatitis (%)	8.0 ± 3.0^†^	10.0 ± 5.0^†^	20.0 ± 5.0
Bilateral prostate-vesiculitis (%)	30.0 ± 5.0^†^	30.0 ± 5.0^†^	5.0 ± 5.0
Bilateral prostate-vesiculitis-epididymitis (%)	30.0 ± 5.0^*∗*†^	18.0 ± 5.0^†^	5.0 ± 5.0

Prevalence of fibrosclerotic versus hypertrophic-congestive variant of MAGI			
Fibrosclerotic MAGI (%)	70.0 ± 10.0^*∗*†^	48.0 ± 5.0^†^	15.0 ± 10.0
Hypertrophic-congestive MAGI (%)	30.0 ± 10.0^*∗*†^	52.0 ± 5.0^†^	85.0 ± 10.0

HPV: human *papilloma virus*. ^*∗*^ *p* < 0.05 versus patients with MAGI *Chlamydia trachomatis*-positive; ^†^ *p* < 0.05 versus patients with inflammatory MAGI.

**Table 4:** Additional criteria for the clinical management of patients with male accessory gland infection/inflammation (MAGI).

What to do?	When?
*Papilloma virus* detection	(i) All patients with MAGI
	(ii) Bilateral prostate-vesicular-epididymitis
	(iii) Fibrosclerotic variant of MAGI
	(iv) Periurethral prostate sign

Ultrasound evaluation	(i) To define the diagnostic category of MAGI (prostatitis alone and prostate-vesiculitis versus prostate-vesicular-epididymitis)
	(ii) To determine if MAGI is monolateral or bilateral
	(iii) For a prognostic evaluation (before treatment)
	(iv) To evaluate the persistence (after treatment)
	(v) To evaluate the eradication (after treatment)
	(vi) For a differential diagnosis (viral versus other forms)

Seminal fluid leukocyte measurement by CD45 antibody staining (relatively expensive, nonroutine investigation).	(i) To evaluate the persistence (after treatment)
	(ii) To evaluate the eradication (after treatment)
	(iii) Viral etiology (*papilloma virus*)
